# Clinical value of artificial intelligence 3D echocardiography in evaluating left atrial volume and pulmonary vein structure in patients with atrial fibrillation

**DOI:** 10.1016/j.clinsp.2024.100487

**Published:** 2024-09-14

**Authors:** Xiaomin Yang, Shujun He, Yang Pang, Kun Rong

**Affiliations:** a Department of Cardiovascular Medicine, Xinhua Hospital Affiliated to Shanghai Jiao Tong University School of Medicine, Shanghai City, China; b Department of Ultrasound, Ezhou Central Hospital, Ezhou City, Hubei Province, China; c Department of Cardiovascular Medicine, Shanghai Changzheng Hospital (The Second Affiliated Hospital of Naval Medical University), Shanghai City, China; d Department of Ultrasound Diagnosis, Qingdao Special Servicemen Recuperation Center of PLA Navy, Qingdao City, Shandong Province, China

**Keywords:** Atrial Fibrillation, Left atrial volume, Pulmonary vein structure, Three dimensional echocardiography, Clinical value

## Abstract

**Objective:**

To explore the clinical value of 3D Echocardiography (3DE) in evaluating the changes of left atrial volume and pulmonary vein structure in patients with Atrial Fibrillation (AF).

**Methods:**

Clinical data were collected from 54 AF patients. Left Atrial Anteroposterior Diameter (LADap), Left Atrial left and right Diameter (LADml), and Left Atrial upper and lower Diameter (LADsi) were measured; the maximum Left Atrial Volume (LAVmax), minimum Left Atrial Volume (LAVmin), left atrial presystolic volume (LAVpre), and Cross-Sectional Area (CSA) of each pulmonary vein were analyzed. Passive Ejection Fraction (LAPEF) was calculated. The differences in left atrial volume and pulmonary vein structure between patients with AF and healthy people were compared, and the correlation between the indexes was analyzed. The diagnostic value of the above indicators for AF patients was analyzed.

**Results:**

LADap, LADml, LADsi, LAVmax, LAVmin, LAVpre, LAPEF, LSPV CSA, LIPV CSA, RSPV CSA, and RIPV CSA of AF patients were significantly higher. There was a significant positive correlation between left atrial diameter and pulmonary vein structure. There was a significant positive correlation between left atrial volume and pulmonary vein structure. There was a negative correlation between LAPEF and pulmonary vein structure. LADap, LADml, LADsi, LAVmax, LAVmin, LAVpre, LAPEF, LSPV CSA, LIPV CSA, RSPV CSA, and RIPV CSA had a diagnostic value for AF patients.

**Conclusion:**

3DE is applicable for evaluating left atrial volume and pulmonary vein structure in patients with AF.

## Highlights

• The ADap, LADml, LADsi, LAVmax, LAVmin, LAVpre, LAPEF, LSPV CSA, LIPV CSA, RSPV CSA, RIPV CSA of AF patients were significantly higher.
• There was a significant positive correlation between left atrial diameter and pulmonary vein structure.
• There was a significant positive correlation between left atrial volume and pulmonary vein structure.
• There was a negative correlation between left atrial active ejection fraction and pulmonary vein structure.
• LADap, LADml, LADsi, LAVmax, LAVmin, LAVpre, LAPEF, LSPV CSA, LIPV CSA, RSPV CSA, RIPV CSA have diagnostic value for AF patients.

## Introduction

Atrioventricular Arrhythmia (AF) is one of the most common types of arrhythmias in clinical practice. AF is primarily characterized by absolute ventricular arrhythmia and variations in heart sound intensity.^1^ According to statistics, there are about 33 million AF patients worldwide.^2^^,^^3^ In China, about 10 million people suffer from AF, and the incidence of AF is about 0.77 %.^4^^,^^5^ AF can be categorized as paroxysmal, persistent, and permanent^6^ and is associated with common cardiovascular and cerebrovascular diseases such as heart failure, stroke, and pulmonary embolism, and the risk of the disease increases significantly with age, resulting in a relatively heavy social and economic burden.^7^^,^^8^ It has been confirmed that ectopic pacing points in the Pulmonary Vein (PV) are a major trigger for AF, which further leads to remodeling of the left atrium and PVs. Therefore it is important to quickly and accurately assess the structure and function of the left atrium and PV.^9^^,^^10^

Two-Dimensional Echocardiography (ECG) is currently the main method used in clinics to evaluate AF patients' functional status. Due to advanced age, lung gas interference, and patient body size, it is often difficult to acquire clear two-dimensional ultrasound images, impacting the accuracy of the structural and functional assessment of the LA and PV.^11^ In contrast, Three-Dimensional ECG (3DE) can well display the endocardial boundary, improve the clarity of ultrasound images in patients with AF, and more accurately evaluate cardiac function.^12^ Early 3DE is based on manual scanning or mechanical sensors to collect images, so this method is cumbersome and time-consuming, and cannot be applied to daily clinical work.

Artificial Intelligence (AI) is a technology that attempts to automate tasks completed by humans. Its research progress in image processing stems from the vigorous development of its convolutional neural network.^13^. In recent years, AI has been continuously applied to medical fields, including static image analysis such as X-Ray, Computed Tomography (CT), and Cardiac Magnetic Resonance imaging (CMR). Moreover, AI technology is also applied to dynamic image analysis.^14^^,^^15^ A variety of 3DE software packages have been developed to assist image analysis with AI techniques to assess the structure and function of the heart and veins.^16^, ^17^, ^18^ In line with this, this study was to explore the clinical value of AI 3DE in evaluating the changes in left atrial volume and PV structure in patients with AF.

## Materials and methods

### General information

54 AF patients hospitalized in Xinhua Hospital Affiliated with Shanghai Jiao Tong University School of Medicine from October 2020 to October 2022 were selected. Inclusion criteria: (1) Patients meeting the diagnostic criteria of AF management guidelines issued by the American College of Cardiology (ACC), American Heart Association (AHA), the Heart Rhythm Society (HRS), and Society of Thoracic Surgeons (STS); (2) Patients diagnosed as AF by ECG or dynamic ECG; (3) Age > 18-years-old; (4) Patients with normal mentality who can cooperate with the investigation and analysis. Exclusion criteria: (1) Congenital heart disease; (2) Heart valve disease; (3) Coronary atherosclerotic heart disease; (4) Previous cardiac surgery history; (5) AF caused by hyperthyroidism or mental factors; (6) Other diseases unsuitable for 3DE. 26 healthy people who came to the Xinhua Hospital Affiliated To Shanghai Jiao Tong University School of Medicine for physical examination at the same time were selected. Clinical data of all subjects were collected, including age, sex, Body Mass Index (BMI), Systolic Blood Pressure (SBP), Diastolic Blood Pressure (DBP), history of smoking, and complications such as hypertension, diabetes, and coronary heart disease. The study was approved by the Xinhua Hospital Affiliated with Shanghai Jiao Tong University School of Medicine ethics committee (n° 201903S10). All patients signed the consent form. This study follows the STROBE statement.

### Detection of two-dimensional 3DE

SIEMEMS ACUSON SC2000 EPIQ 7C color Doppler ultrasound diagnostic instrument, equipped with S5-1 two-dimensional probe and 4Z1c 3D probe, with a frequency of 1‒5 MHz, was used for analysis with Syngo Via Q-LAB quantitative analysis software. ECGs were routinely connected to subjects lying on their left side and breathing calmly. The Left Atrial Anteroposterior Diameter (LADap), Left Atrial Left and Right Diameter (LADml), and Left Atrial Upper and Lower Diameter (LADsi) were measured with S5-1 two-dimensional probe. With the real-time 3D ultrasound full-volume mode, the X5-1 3D probe was used to collect images of three consecutive cardiac cycles from the apical four-chamber view. The images were imported into the Q-LAB workstation and analyzed by the 3DQA mode. The maximum Left Atrial Volume (LAVmax), minimum Left Atrial Volume (LAVmin), and Left Atrial Presystolic Volume (LAVpre) were analyzed. Left Atrial Appendage Ejection Fraction (LAAEF) was calculated as (LAVmax LAVmin)/LAVpre. Left Atrial Passive Ejection Fraction (LAPEF) was measured as (LAVmax LAVmin)/LAVmin. The position and changes of each PV were confirmed according to the coronal, sagittal, and axial 3D images. The maximum Cross-Sectional Area (CSA) of each PV in the sagittal plane perpendicular to the blood flow direction was analyzed, including the CSA of the Left Superior PV (LSPV CSA), the CSA of the Left Inferior PV (LIPV CSA), the CSA of the Right Superior PV (RSPV CSA), and the CSA of the Right Inferior PV (RIPV CSA).

### Statistical analysis

The measurement data were expressed as mean ± standard deviation. Comparisons between the two groups should be made using the *t*-test if the measurements conform to a normal distribution or the Wilcoxon rank sum test if they do not conform to a normal distribution. The counting data were expressed in cases or percentages, and the Chi-Square test or Fisher test was used; *p* < 0.05 indicates a statistical difference.

## Results

### Comparison of general data

Of the 54 AF patients, 31 were male (57.41 %) and 23 were female (42.59 %); The average age was (60.91 ± 11.38) years old; BMI was (23.41 ± 1.17) kg/m^2^, systolic blood pressure was (129.54 ± 9.97) mmHg, and diastolic blood pressure was (71.74 ± 5.24) mmHg; 32 cases (59.26 %) had a history of smoking, 10 cases (18.52 %) had a history of hypertension, 5 cases (44.44 %) had a history of diabetes, and 27 cases (50.00 %) had a history of coronary heart disease. In all 26 healthy subjects, 14 males (53.85 %) and 12 females (46.15 %) were included; The average age was (60.38 ± 11.59) years old; BMI was (23.52 ± 0.90) kg/m^2^, systolic blood pressure was (129.85 ± 8.40) mmHg, and diastolic blood pressure was (71.77 ± 5.73) mmHg. There were 10 cases (38.46 %) with smoking history, 8 cases (19.23 %) with hypertension history, 11 cases (42.31 %) with diabetes history, and 8 cases (30.77 %) with coronary heart disease history. There was no statistical difference in general data between the two groups (*p* > 0.05) Table 1.

**Table 1:** Comparison of general data of subjects.

Item	AF group (*n* = 54)	NC group (*n* = 26)	p-value
Male [n (%)]	31/57.41 %	14/53.85 %	0.7636
Age (years)	60.91 ± 11.38	60.38 ± 11.59	0.8488
BMI (kg/m^2^)	23.41 ± 1.17	23.52 ± 0.90	0.6788
SBP (mmHg)	129.54 ± 9.97	129.85 ± 8.40	0.8919
DBP (mmHg)	71.74 ± 5.24	71.77 ± 5.73	0.9824
Smoking history [n (%)]	32/59.26 %	10/38.46 %	0.0810
Hypertension history [n (%)]	10/18.52 %	5/19.23 %	0.9391
Diabetes history [n (%)]	24/44.44 %	11/42.31 %	0.8568
History of coronary heart disease [n (%)]	27/50.00 %	8/30.77 %	0.1044

### Left atrial volume comparison

**Fig. 1:**
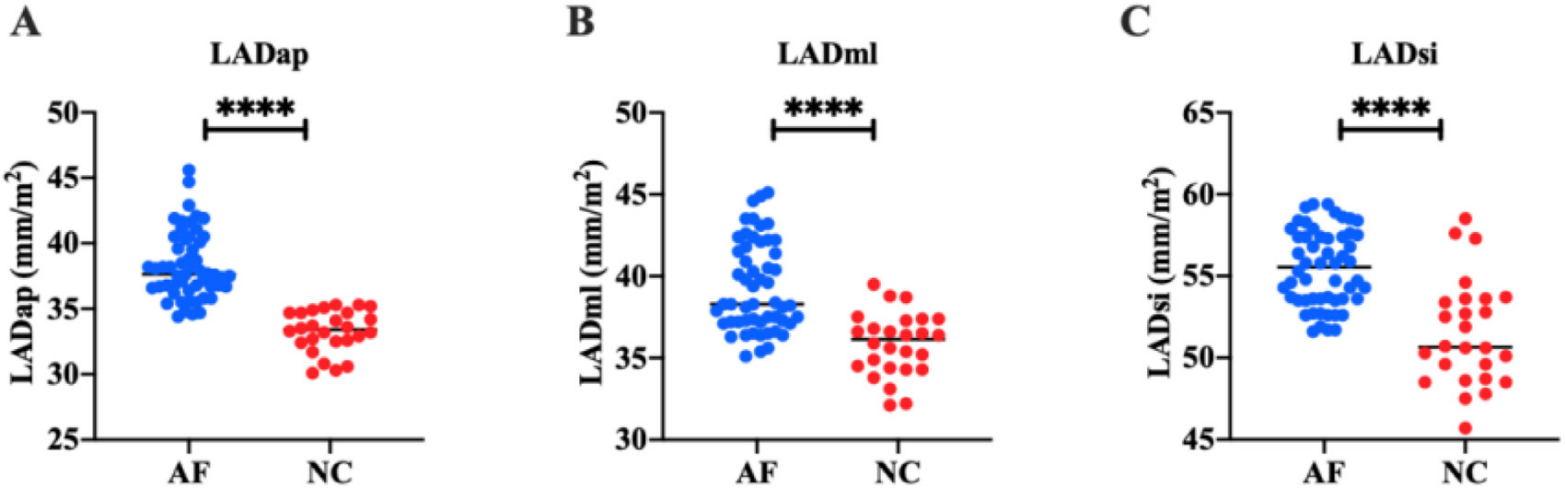
Left atrial diameter. (A‒C) Results of LADap (A), LADml (B), and LADsi (C). **** *p* < 0.0001.

**Fig. 2:**
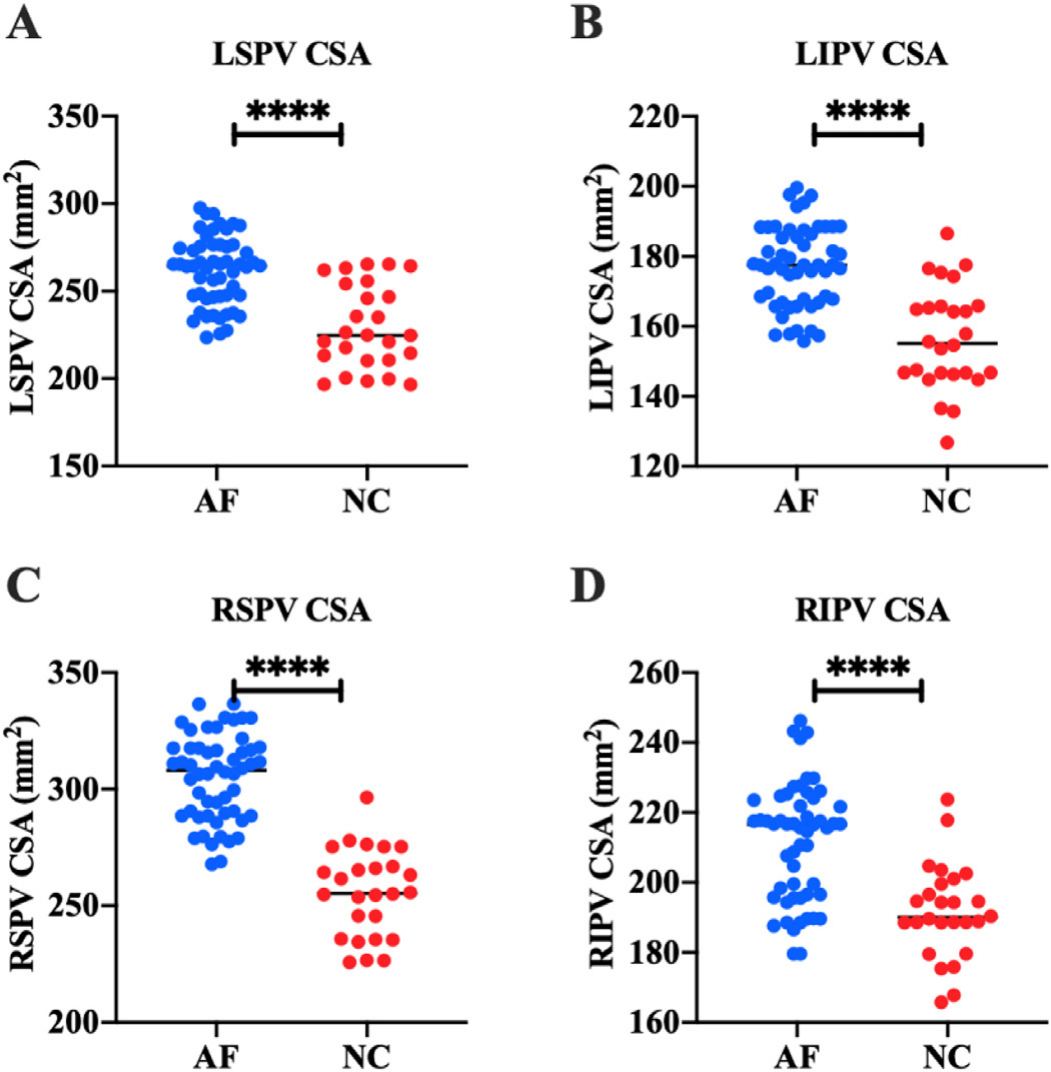
Pulmonary vein structure. (A‒D) Results of LSPV CSA (A), LIPV CSA (B), RSPV CSA (C), and RIPV CSA (D). ****p < 0.0001.

**Fig. 3:**
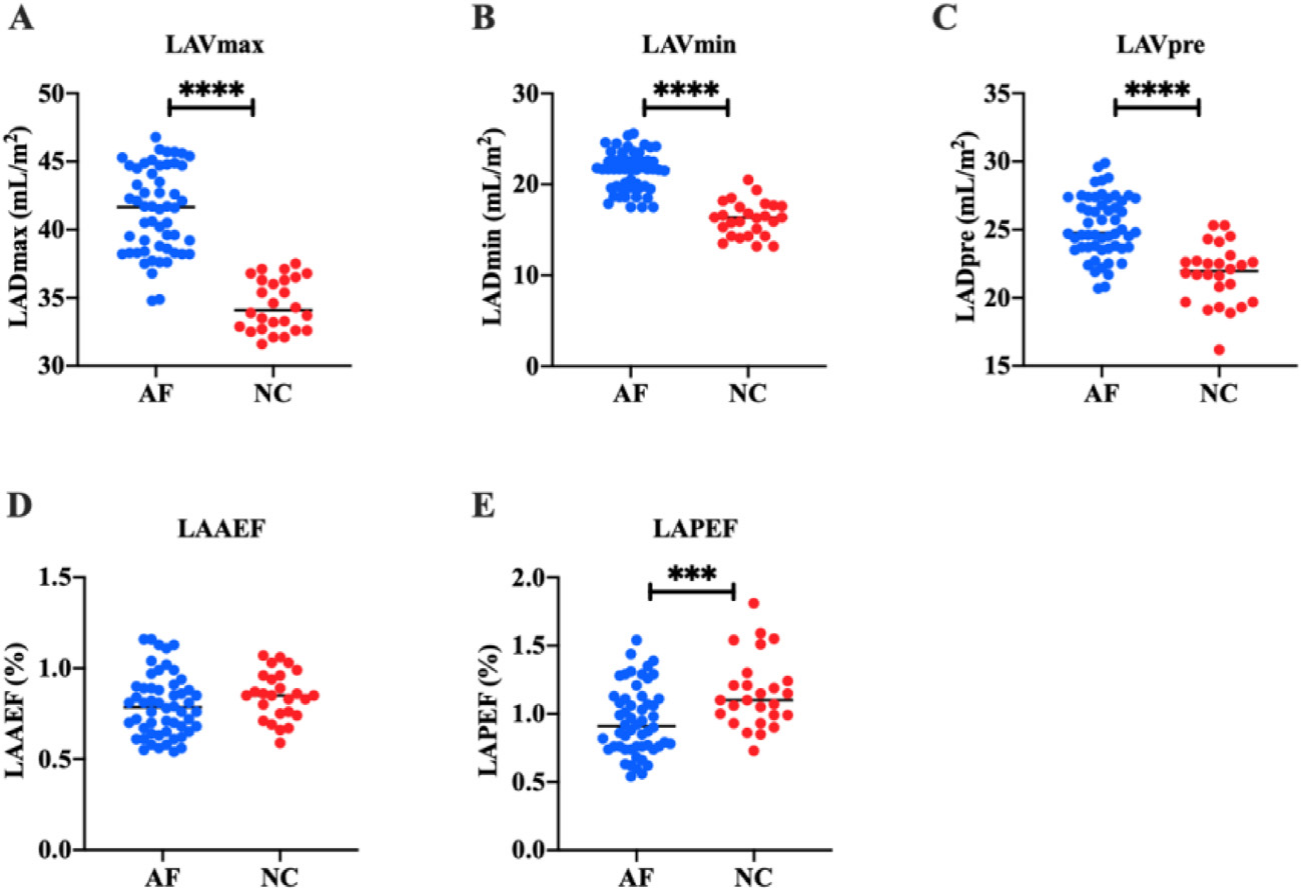
Left atrial volume. (A‒D) Results of LAVmax (A), LAVmin, (B) LAVpre (C), LAAEF (D), and LAPEF (E). *** *p* < 0.001, **** *p* < 0.0001.

In order to study the left atrial function of AF patients, the authors measured the left atrial volume of all subjects by two-dimensional and 3DE. Two-dimensional ECG showed that in the AF group, LADap was (38.31 ± 2.58) mm, LADml was (39.34 ± 2.72) mm, and LADsi was (55.46 ± 2.33) mm. In the NC group, LADap was (33.28 ± 1.59) mm, LADml was (35.83 ± 1.92) mm, and LADsi was (51.50 ± 3.23) mm. These results suggest that LADap, LADsi, and LADml of AF patients are significantly higher than those of healthy people (*p* < 0.0001) (Fig. 1). 3DE showed that in AF group, LAVmax was (41.36 ± 3.13) mL/m^2^, LAVmin was (21.44 ± 2.14) mL/m^2^, and LAVpre was (25.24 ± 2.23) mL/m^2^. In the NC group, LAVmax was (34.49 ± 1.89) mL/m^2^, LAVmin was (16.20 ± 1.90) mL/m^2^, and LAVpre was (21.72 ± 2.18) mL/m^2^. LAAEF and LAPEF were calculated. In the AF group, LAAEF was (0.80 ± 0.17) % and LAPEF was (0.95±0.25) %; LAAEF and LAPEF in the NC group were (0.85 ± 0.13) % and (1.15 ± 0.26) %, respectively. These results suggest that LAVmax, LAVmin, and LAVpre of AF patients are significantly higher than those of healthy people (*p* < 0.0001) (Fig. 2), and LAPEF is lower than that of healthy people (*p* < 0.001) (Fig. 3).

### Comparison of PV structure

The authors detected the structure of PVs in all subjects by 3DE. The results showed that in the AF group, LSPV CSA was (261.44 ± 19.53) mm^2^, LIPV CSA was (177.15 ± 11.53) mm^2^, RSPV CSA was (304.87 ± 18.39) mm^2^, and RIPV CSA was (213.46 ± 22.89) mm^2^. In the NC group, LSPV CSA was (229.63 ± 23.94) mm^2^, LIPV CSA was (156.60 ± 14.73) mm^2^, RSPV CSA was (255.74 ± 18.62) mm^2^, and RIPV CSA was (191.64 ± 13.34) mm^2^. These results suggest that LSPV CSA, LIPV CSA, RSPV CSA, and RIPV CSA in AF patients are significantly higher than those in healthy people (*p* < 0.0001) (Fig. 2).

### Correlation analysis of left atrial volume and PV structure

**Fig. 4:**
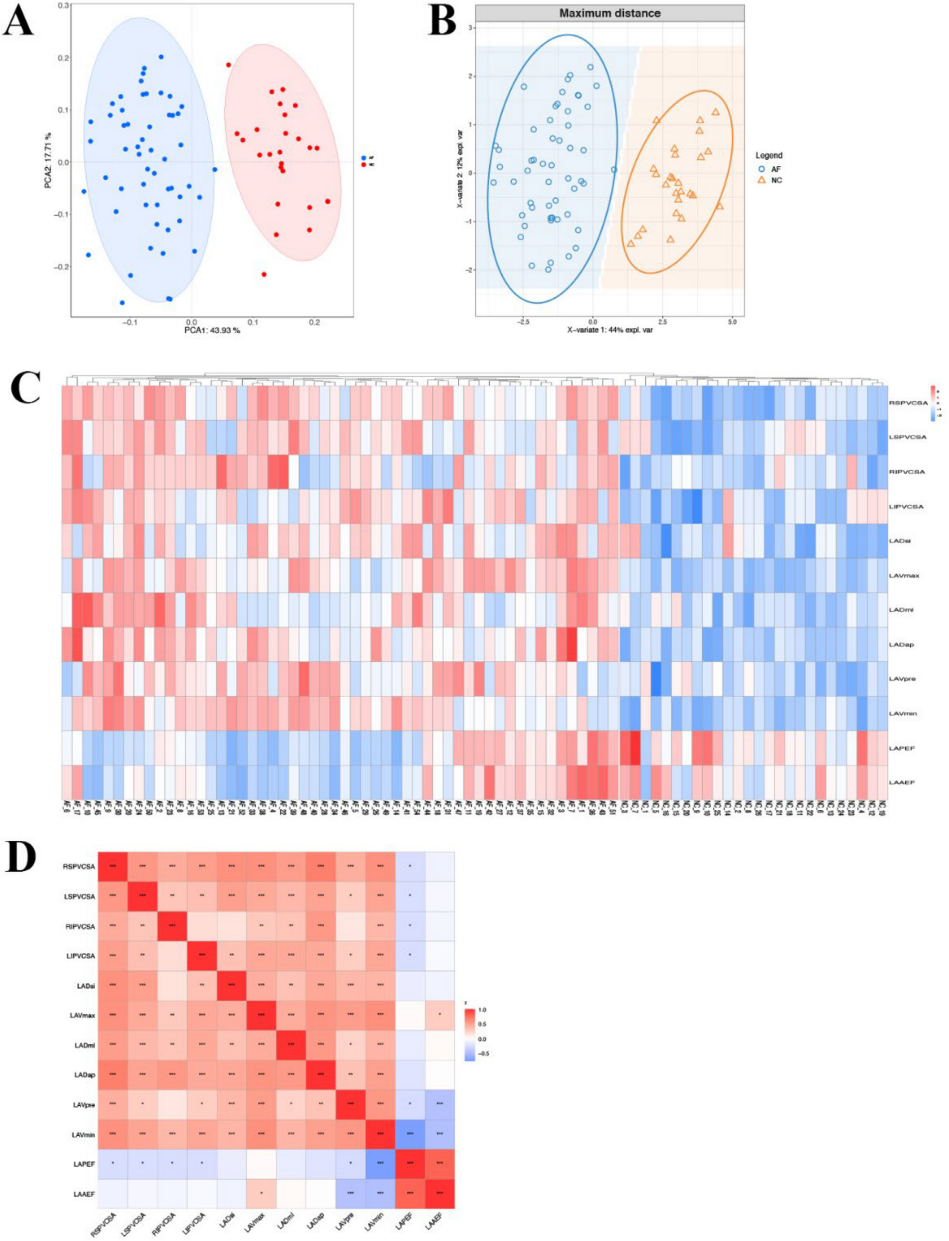
Expression pattern of indexes related to left atrial volume and pulmonary vein structure in subjects. (A) PCA analysis; (B) PLS-DA analysis; (C) Cluster thermogram analysis; (D) Correlation analysis. *p < 0.05, **p < 0.01, ***p < 0.001.

PCA analysis showed that the samples of AF patients and healthy people were separated among PC1 dimension groups (Fig. 4A). At the same time, PLS-DA discriminant analysis based on indicators showed that AF patients and healthy people tended to gather in the sample group and tended to be dispersed between groups (Fig. 4B). Cluster analysis heat map shows that AF patients and healthy people were obviously separated, suggesting that left atrial volume related indicators (LADap, LADml, LADsi, LAVmax, LAVmin, LAVpre, LAAEF, LAPEF) and PV structure related indicators (LSPV CSA, LIPV CSA, RSPV CSA, RIPV CSA) can distinguish AF patients and healthy people (Fig. 4C). Further correlation analysis was carried out, showing that the related indexes of left atrial diameter and PV structure were significantly positively correlated (*p* < 0.01, *p* < 0.001), and the related indexes of left atrial volume and PV structure were significantly positively correlated (*p* < 0.05, *p* < 0.01, *p* < 0.001), and there was a negative correlation between LAAEF and the relevant indicators of PV structure (*p* < 0.05) (Fig. 4D).

### Diagnostic value of left atrial volume in evaluating AF

**Fig. 5:**
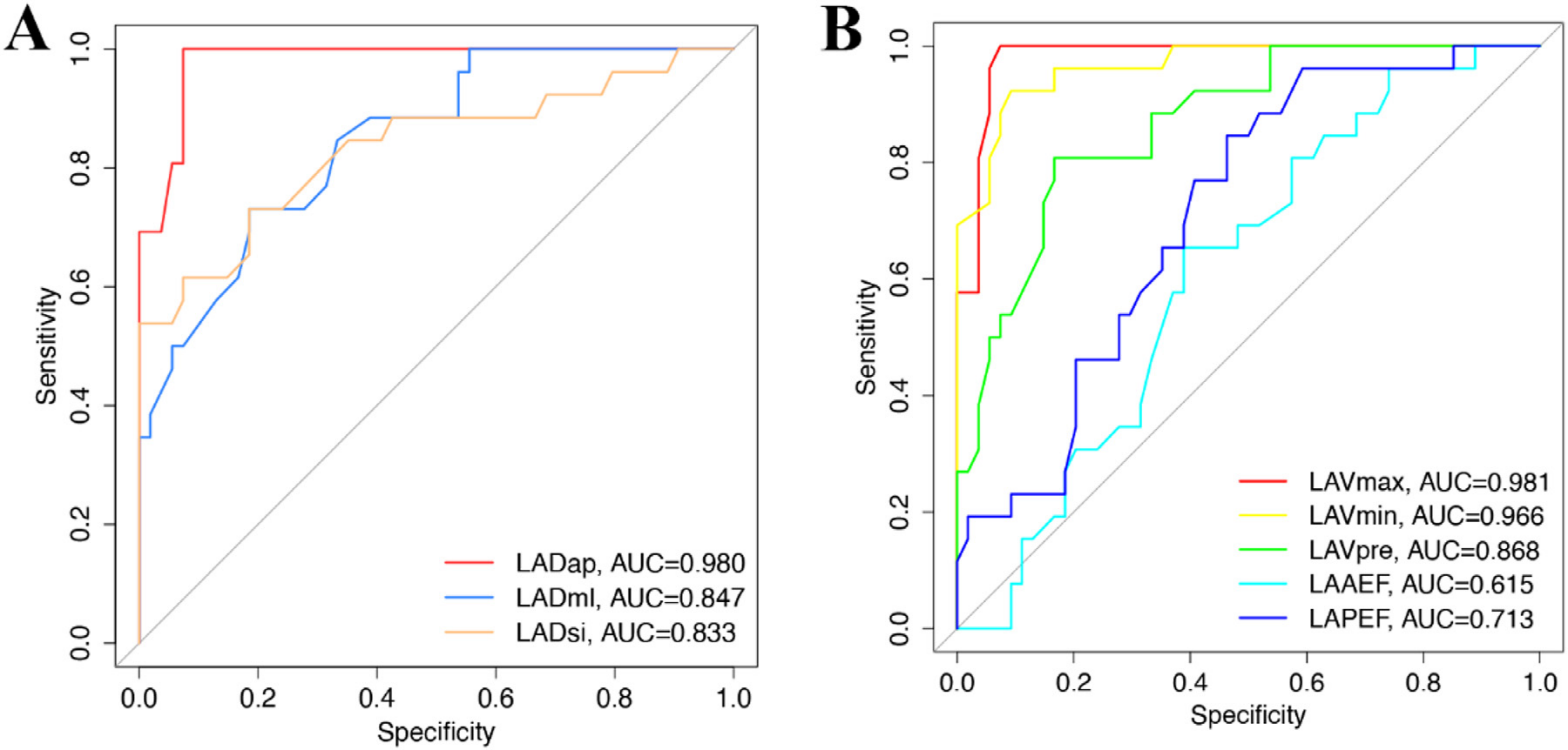
Diagnostic value of left atrial volume index. (A) Diagnostic value of indexes related to left atrial diameter; (B) Diagnostic value of left atrial volume related indexes.

The diagnostic value of left atrial volume was evaluated by ROC. The AUC of LADap, LADml, and LADsi was 0.980 (95 % CI 0.9575‒1.000; *p* < 0.0001), 0.847 (95 % CI 0.7608‒0.9336; *p* < 0.0001), and 0.833 (95 % CI 0.7274‒0.9385; *p* < 0.0001), respectively (Fig. 5A). The AUC of LAVmax, LAVmin, LAVpre, LAAEF, LAPEF was 0.981 (95 % CI 0.9569‒1.000; *p* < 0.0001), 0.966 (95 % CI 0.9301‒1.000; *p* < 0.0001), 0.868 (95 % CI 0.7883‒0.9474; *p* < 0.0001), 0.615 (95 % CI 0.4905‒0.7389), and 0.713 (95 % CI 0.5989‒0.8263; *p* < 0.01), respectively (Fig. 5B). These results suggest that LADap, LADml, LADsi, LAVmax, LAVmin, LAVpre, and LAPEF have a diagnostic value for AF patients.

### Diagnostic value of PV structure evaluation on AF

**Fig. 6:**
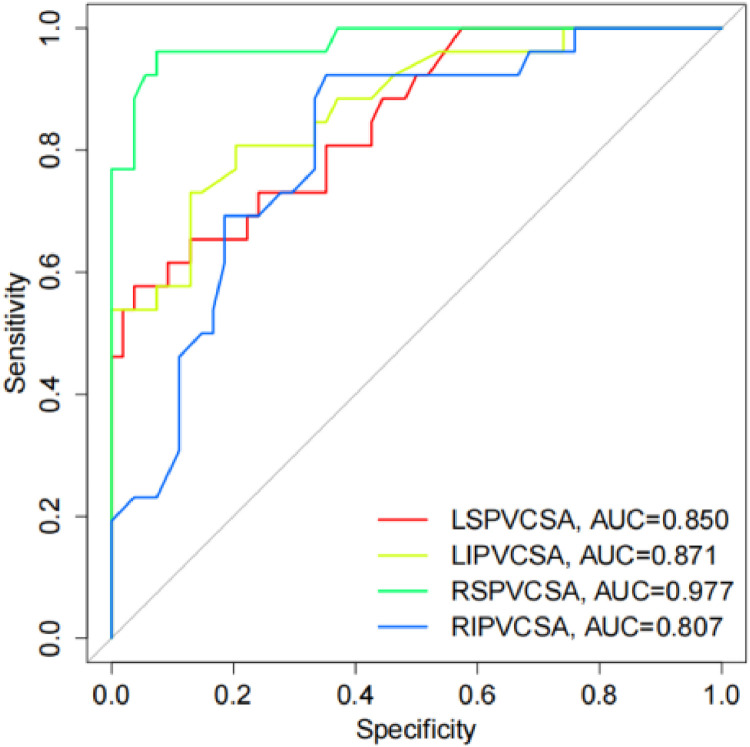
The diagnostic value of PV structural indicators in patients with AF.

The diagnostic value of PV structure was evaluated by ROC. The results showed that the AUC of LSPV CSA, LIPV CSA, RSPV CSA, and RIPV CSA was 0.850 (95 % CI 0.7614‒0.9380; *p* < 0.0001), 0.871 (95 % CI 0.7871‒0.9551; *p* < 0.0001), 0.977 (95 % CI 0.9468‒1.000; *p* < 0.0001), and 0.807 (95 % CI 0.7087‒0.9053; *p* < 0.0001), respectively (Fig. 6), indicating that LSPV CSA, LIPV CSA, RSPV CSA, and RIPV CSA have a diagnostic value for AF patients.

## Discussion

AF is a common arrhythmia in clinical practice.^19^ On the one hand, AF increases the afterload on the left atrium, causes mechanical and electrical remodeling of the left atrium, and ultimately enlarges the diameter of the left atrium and decreases its function.^20^ After AF is converted and maintained in sinus rhythm, myocardial fibrosis is improved, and atrial remodeling is reversed, which is conducive to inhibiting remodeling and improving cardiac function.^21^^,^^22^ This is because the effect of AF on cardiac function is related to its reduction of left atrial filling to the left ventricle and normal stroke volume.^23^ On the other hand, the adverse prognosis of patients with AF is related to thromboembolism events caused by thrombosis shedding.^24^ The traditional diagnostic methods of AF mainly include cardiac examination and electrocardiogram. The 12 lead ECG is the gold standard for diagnosing AF according to the RR interval and P wave.^25^^,^^26^ The sharp increase in the number of AF patients makes it sometimes difficult for doctors to diagnose a large number of ECGs in time. Paroxysmal AF may also lead to missed diagnosis due to failure to record the onset of AF in an ordinary 12-lead or 24 h dynamic ECG. Implantable ECG event recorders can extend the monitoring time to 3 years, but their high price also brings a burden to clinical work.^27^ In the absence of timely diagnosis, AF patients will suffer severe thromboembolism events. The need for a non-invasive, cheap, and simple method of monitoring AF is urgent, both for doctors and patients.

3D ultrasound technology integrates a matrix probe, a high-channel data processing system, and a 3D spatial positioning system. Research shows that 3DE has been initially applied to assess the structure and function of the left atrium in patients with AF.^28^^,^^29^ According to the three different stages of the normal cardiac cycle, the left atrial function can be divided into storage function, ductal function and auxiliary pump function. Storage function refers to how well the left atrium fills with blood returned by the PV during systole, a function that relates primarily to compliance of the left atrium. In early diastole, the ductal function reflects how well the left atrium delivers blood to the left ventricle, and it is affected by both left atrium compliance and left ventricle diastolic function. The auxiliary pump function reflects the ability of the left atrium to actively pump blood to the left ventricle in the late diastole and is related to the left atrial systolic force and the left atrial pre and post-load. The indexes related to the Left Atrium Volume (LADap, LADml, LADsi, LAVmax, LAVmin, LAVpre, LAAEF, LAPEF) can well reflect the left atrium function.^30^, ^31^, ^32^ A study involving 47 patients with AF and 25 healthy volunteers showed that LAVmax and LAVmin in AF patients increased, while LATEF decreased. Six months after ablation, LAVmax and LAVmin decreased significantly, and LATEF increased significantly.^33^ The study also found that there was no significant change in all parameters 3 days after the operation, so it was speculated that after ablation, the left atrium may be stunned for a short period of time, and gradually recover after six months.^33^ Another study involving 62 AF patients who successfully received ablation showed that LAVmax and LAVpre decreased significantly, LAVmin did not change significantly, LAAEF and LATEF increased significantly, and LAPEF did not change significantly 3 months after operation.^34^ This study found that compared with healthy people, the left atrial volume of AF patients increased and left atrial remodeling existed. At the same time, LADml, LADsi, LAVmax, LAVmin, LAVpre and LAPEF were potential clinical indicators to predict AF.

A large number of previous studies have confirmed that most of the abnormal trigger foci of pulmonary venous origin in atrial fibrillation are located in the upper segment of the PVs.^35^^,^^36^ There are four PVs in the human body, two on the left and two on the left, which are respectively the left upper PV, the left lower PV, the right upper PV, and the right lower PV. The left upper PV and the left lower PV converge into a trunk and flow into the left atrium, or three right PVs from three lobes flow into the left atrium respectively. The study shows that the variation rate of PVs in AF patients is between 20 %‒40 %.^37^^,^^38^ Patients with atrial fibrillation have the most variable pulmonary venous coaptation, with PV orifice diameters being largest in systole and smallest in diastole. Preliminary results showed that 3DE can dynamically display the shape and displacement of each PV and the relationship of the PVs to the left atrium in most patients in a multidirectional and multifaceted manner, as well as measure each PV. Heart rate had no significant impact on the imaging quality.^39^^,^^40^ This study found that the structural indicators of PVs in patients with AF were significantly higher than those in healthy people, and LSPV CSA, LIPV CSA, RSPV CSA and RIPV CSA were also potential indicators to predict AF.

In conclusion, the indexes of left atrial volume in patients with AF are different from those in healthy people, and the indexes of PV structure are also significantly higher than those in healthy people. The indexes related to left atrial volume (LADap, LADml, LADsi, LAVmax, LAVmin, LAVpre) are significantly positively correlated with the indexes related to PV structure, and LAPEF is negatively correlated with the indexes related to PV structure. At the same time, the relevant indexes of left atrial volume and PV structure are potential indexes to predict AF. AI-3DE technology is expected to become a clinical diagnostic tool to evaluate the changes in left atrial volume and PV structure in patients with AF.

## Availability of data and materials

The data and materials used to support the findings of this study are available from the corresponding author.

## Ethics statement

The study was approved by the Xinhua Hospital Affiliated to the Shanghai Jiao Tong University School of Medicine ethics committee (n° 201903S10), and performed in accordance with The Declaration of Helsinki. Written informed consent was obtained from all patients prior to the study start.

## Authors’ contributions

Xiaomin Yang and Shujun He designed the research study. Yang Pang and Kun Rong performed the research. Shujun He and Yang Pang provided help and advice on the experiments. Xiaomin Yang and Kun Rong analyzed the data. Xiaomin Yang and Shujun He wrote the manuscript. Kun Rong reviewed and edited the manuscript. All authors contributed to editorial changes in the manuscript. All authors read and approved the final manuscript.

## Funding

Not applicable.

## Declaration of competing interest

The authors declare no conflicts of interest.
